# Routine colonoscopy may be needed for uncomplicated acute right colonic diverticulitis

**DOI:** 10.1186/s12876-021-01672-1

**Published:** 2021-02-27

**Authors:** Kil-yong Lee, Jaeim Lee, Youn Young Park, Seong Taek Oh

**Affiliations:** Division of Coloproctology, Department of Surgery, Uijeongbu St. Mary’s Hospital, College of Medicine, The Catholic University of Korea, 271, Cheonbo-ro, Uijeongbu, Gyeonggi-do 11765 Korea

**Keywords:** Diverticulitis, Acute diverticulitis, Colonic evaluation, Endoscopy, Colonoscopy, Colonic neoplasia

## Abstract

**Background:**

Routine colonoscopy is recommended to determine the coexistence of colon cancer after medical treatment for colon diverticulitis. However, in the case of uncomplicated diverticulitis diagnosed by computed tomography, the clinical relevance of routine follow-up colonoscopy has recently been debated. Yet, the role of follow-up colonoscopy for right colon diverticulitis, which tends to develop at a younger age than left colon diverticulitis, has not been specifically evaluated. Therefore, we aimed to evaluate the incidence of right colon cancer or colonic adenomatous polyps, detected by routine colonoscopy, after conservative management of acute uncomplicated right colon diverticulitis.

**Methods:**

Patients with uncomplicated right colon diverticulitis (modified Hinchey stage Ia) diagnosed by computed tomography imaging, between 2011 and 2017, and who underwent follow-up colonoscopy surveillance after treatment were included. The primary outcome was the incidence of colon cancer, with the detection rate of adenoma being the secondary outcome. Information for analysis was retrieved retrospectively from patients’ medical records.

**Results:**

The study group included 330 consecutive patients, with a mean age of 41.9 years, and 51.9% being men. For the primary outcome, the rate of colon cancer on follow-up colonoscopy was 0.3% (1/330 cases). The rate of adenoma detection was 20.9% (69/330 cases) and advanced adenoma (> 10 mm in diameter; or exhibiting a > 25% villous component or severe dysplasia), including colon cancer, was observed in 9 patients (2.7%).

**Conclusions:**

In patients with acute uncomplicated right colonic diverticulitis, routine colonoscopy after conservative treatment may be necessary because although the colon cancer detection rate is low, it is possible to detect advanced colon adenoma.

## Background

The prevalence of diverticular disease has increased, both the in East and West, due to a lack of dietary fiber intake [1]. In contrast to complicated diverticulitis, which requires surgical treatment, uncomplicated acute diverticulitis can improve with conservative management, with colonoscopy recommended to identify accompanying disease, such as cancer [2]. Yet, a review article reported a low rate of cancer diagnosis during colonoscopy surveillance in patients treated for uncomplicated diverticulitis, with 1 case of cancer among the 67 cases included in the analysis [3]. However, these statistics are based solely on cases with left colon diverticulitis and only small number of patients (67 patients) were included. As such, the likelihood of cancer among patients treated with uncomplicated right colon diverticulitis is unknown. This knowledge, however, would be important as right-sided colon diverticulitis tends to occur at a younger age than left-sided colon diverticulitis, with a low stage Hinchey classification being common on computed tomography (CT) [4]. Generally, the American Cancer Society guideline recommends colonoscopy surveillance to begin at the age of 45 years [5]. Moreover, the onset of colon cancer at a younger age is more likely to occur in the left than right colon [6]. Considering these points, it is unclear how routine colonoscopy evaluation after right colon diverticulitis could be of clinical benefit with regard to diagnostic performance and economic burden.

Of the literature published on the effects of routine colonoscopy after the treatment of uncomplicated colon diverticulitis, few articles have exclusively included patients with right colon diverticulitis. Thus, the aim of our study was to evaluate the incidence of right colon cancer and colonic adenomatous polyps by routine colonoscopy, after conservative management of acute uncomplicated right colon diverticulitis.

## Methods

We retrospectively reviewed the electronic medical records of patients diagnosed with acute right-sided colon diverticulitis, between January 2011 and December 2017, at our hospital.

Patients with acute uncomplicated diverticulitis confirmed by CT, were included. Acute uncomplicated diverticulitis was defined, in accordance with previous studies [7, 8], as the presence of colonic diverticular disease with localized colonic wall thickening and/or stranding of pericolonic fat. CT images with appearances of complicated diverticulitis (defined as the presence of pericolonic or abdominal abscess, localized or free extraluminal gas or contrast, obstruction or fistula formation) or the presence of an associated mass lesion were excluded. Follow-up colonoscopy was performed after improvement in the signs of inflammation corresponding to acute diverticulitis, with the consent of the patient. Exclusion criteria were as follow: complicated diverticulitis; accompanying left colon cancer; patients who underwent emergency surgery; and patients in whom follow-up colonoscopy was not performed or was not consented to. The reasons why a patient did not undergo colonoscopy was investigated based on a previous paper [9].

The following colonoscopy findings were documented: hyperplastic polyp, adenoma (including advanced adenoma), and carcinoma. Patients with polyps detected in the right colon were the focus of our analysis. Advanced adenoma was defined as either an adenoma of 10 mm or greater in diameter and/or more than 25% villous components and/or severe dysplasia [10].

The primary outcome was the detection rate of colon cancer, confirmed by pathological diagnosis, on follow-up colonoscopy. The secondary outcome was the detection rate of hyperplastic polyp and adenoma on follow-up colonoscopy.

### Statistical analysis

Categorical variables were reported as a count (and associated percentage, %), with continuous variables reported as the median and interquartile range (IQR). We analyzed the group with and without colonoscopy to know the characteristics of patients who have not performed the colonoscopy to be sure that the included patients are representative of the entire sample. To compare the group with and without colonoscopy, the chi-squared or Fisher exact test was used for categorical variables, as appropriate. As such, between-group comparisons of continuous variables were evaluated using the Mann–Whitney test. Additionally, the difference in the frequency of polyp detection was compared among patients under 45 years of age and over 45 years of age who underwent colonoscopy using the chi-squared or Fisher exact test.

All analyses were performed using SPSS (version 21, IBM, NY, USA).

## Results

**Fig. 1:**
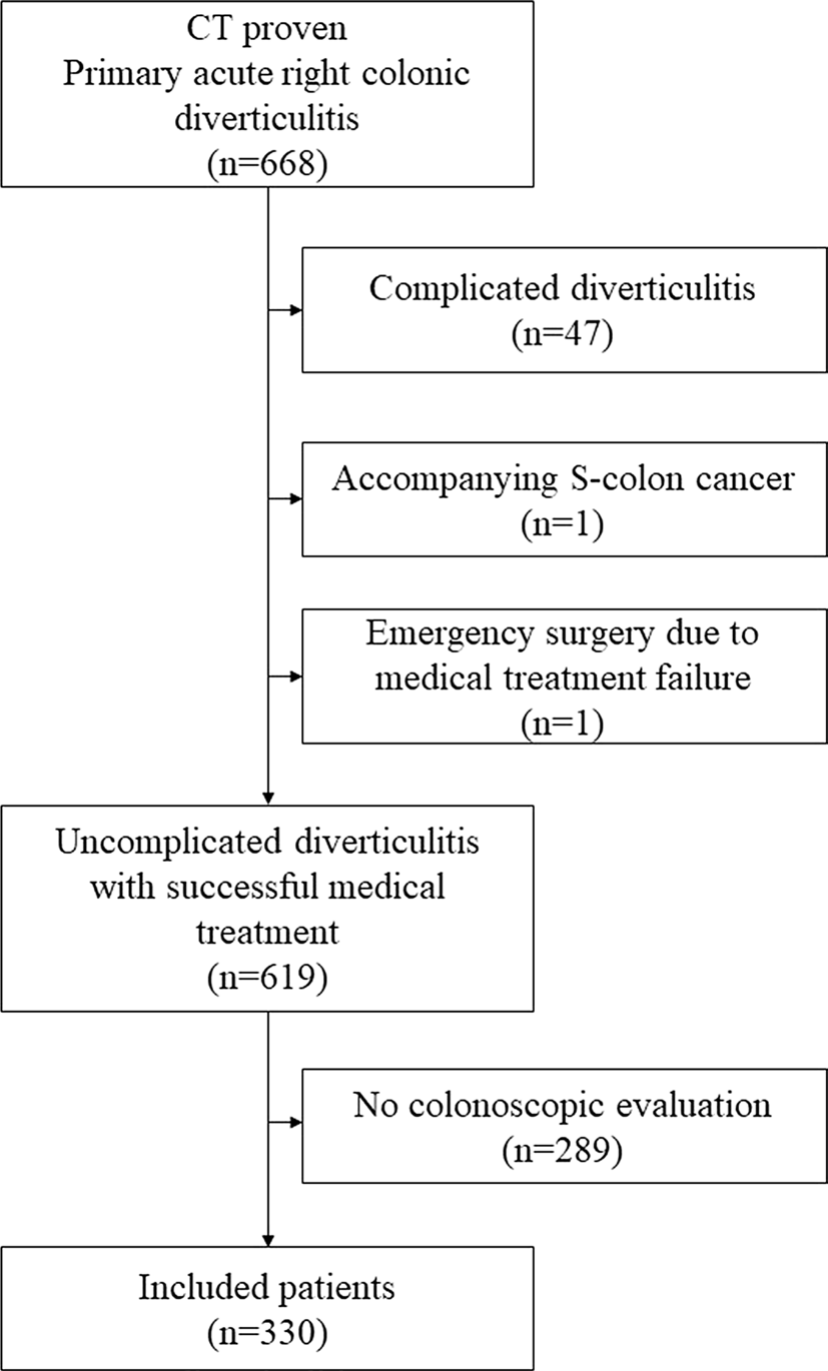
Flow diagram of patient selection

A total of 668 consecutive patients were diagnosed with acute right colon diverticulitis, by CT, over the period of observation of the study. From these, the following patients were excluded from the analysis: 47 who had complicated diverticulitis; 1 with sigmoid colon cancer simultaneously diagnosed on CT imaging; and 1 who underwent emergency surgery because symptoms did not improve. Of the remaining 619 patients, follow-up colonoscopy was performed in 330 (Fig. 1). Follow-up colonoscopy was performed at a median of 32 days (IQR, 25–42 days) after recovery from acute diverticulitis.

Clinical characteristics of patients included in the analysis are reported in Table 1, with salient characteristics as follows: median age, 40 (IQR, 33–49) years; male, 59.7%; and predominant involvement of the cecum (48.3%) and ascending colon (49.9%). With regard to clinical factors, only the length of hospital stay was significantly different between the two groups.

**Table 1:** Clinical characteristics of patients with acute uncomplicated diverticulitis

Variables	Colonoscopy (n = 330)	No colonoscopy (n = 289)	*p* value
Age (years)	40 (34–50)	40 (31–48)	0.168^d^
Age group			0.656^e^
< 45 (years)	209 (63.3%)	188 (65.1%)	
> 45 (years)	121 (36.7%)	101 (34.9%)	
Sex			0.778^e^
Male	195 (59.1%)	174 (60.2%)	
Female	135 (40.9%)	115 (39.8%)	
Height (cm)	167.5 (160.0–173.5)	168.0 (160.4–173)	0.962^d^
BMI (kg/m^2^)	23.8 (21.7–26.3)	23.4 (21.6–25.9)	0.135^d^
Duration of hospital stay (days)	4 (4–5)	4 (3–5)	0.010^d^
Social history			
Smoking	142 (43%)^a^	139 (48.1%)	0.303^e^
Alcohol	137 (42.7%)^b^	110 (38.1%)^c^	0.248^e^
Past medical history			
Hypertension	50 (15.2%)	31 (10.7%)	0.103^e^
Diabetes	14 (4.2%)	12 (4.2%)	0.956^e^
Location of the acute diverticulitis			0.770^e^
Cecum	154 (46.7%)	144 (49.8%)	
Ascending	171 (51.8%)	139 (48.1%)	
Hepatic flexure	3 (0.9%)	3 (1.0%)	
Transverse	2 (0.6%)	3 (1.0%)	
White blood cell count (10^3^/μl)	11.1 (9.2–12.9)	11.5 (9.7–14.0)	0.082^d^
C-reactive protein (mg/dl)	3.5 (1.6–6.5)	3.8 (1.6–7.0)	0.553^d^

Continuous variables are reported as the median (and IQR)

Categorical variables are reported as a count (and percentage, %)

*IQR* interquartile range, *BMI* body mass index

^a^Missing data: n = 1

^b^Missing data: n = 9

^c^Missing data: n = 13

^d^Mann–Whitney test

^e^Chi-squared test

On follow-up colonoscopy, hyperplastic polyps were identified in 30 patients (9.1%) and adenomas in 69 patients (20.9%), with evidence of high-grade dysplasia in 2 of these cases. Villous adenoma was detected in 1 case. An advanced adenoma, including colon cancer, was observed in 9 patients (2.7%), one of whom was diagnosed with ascending colon cancer. Polyps were found in the right colon in 46 of 87 patients with colon polyps (Table 2). Additionally, on biopsy, chronic inflammation of the right colon was confirmed in 13 patients: erythematous mucosal change (n = 5) or ulceration (n = 8). Furthermore, right colonic diverticuli were found in 261 (79.1%) of 330 patients who underwent colonoscopy. There was no incidence of inflammatory bowel disease combined with diverticulosis.

**Table 2:** Results of colonoscopy examination

Variables	Value
Hyperplastic polyp	30 (9.1%)
Adenoma	
Low grade dysplasia	67 (20.3%)
High grade dysplasia	2 (0.6%)
Villous adenoma	1 (0.3%)
Advanced adenoma	9 (2.7%)
> 10 mm in diameter	8 (2.4%)
> 25% villous components	1 (0.3%)
High grade dysplasia	2 (0.6%)
Adenocarcinoma	1 (0.3%)
Right-sided polyp^a^	46 (52.9%)^a^

^a^Number of patients in whom polyps were detected in the right colon among patients with adenoma detected. (total number of patients, 87)

Ascending colon cancer was found in one male patient in his late 40 s at 6 weeks after he originally presented to our emergency department owing to a 2-day history of abdominal pain. CT examination, performed by an expert radiologist, revealed a segmented cecum and thickening of the wall of the ascending colon, with perilesional fat infiltration. He was admitted and treated with a 5-day course of intravenous antibiotics for a presumed diagnosis of uncomplicated right-sided acute diverticulitis. Follow-up colonoscopy was performed 6 weeks after imaging, with a diagnosis of ascending colon cancer confirmed.

In uncomplicated diverticulitis (330 patients) who underwent colonoscopy, recurrence occurred in 30 cases (9.1%) for a median follow-up period of 680 days, and surgery was performed in a total of 5 cases (1.5%) (in 4 cases for recurrent diverticulitis and in 1 case for the aforementioned cancer).

The reasons for lack of follow-up colonoscopy are summarized in Table 3, and main reasons were summarized as follows: 103 patients (35.6%) scheduled colonoscopy, but not yet performed with unknown causes, 86 patients (29.8%) were lost to follow-up, 22 patients (7.8%) had a history of a recent prior colonoscopy; 15 (5.3%) underwent follow-up colonoscopy at another hospital; and 10 (3.5%) were followed up using colon barium study at the patient’s request.

**Table 3:** Reasons for patients to not undergo colonoscopy

Reasons	Number (%)
Prior colonoscopy ≤ 12 months prior	15 (5.2%)
12 < Prior colonoscopy ≤ 24 months	3 (1.0%)
24 < Prior colonoscopy ≤ 36 months	1 (0.3%)
36 < Prior colonoscopy ≤ 60 months	2 (0.7%)
60 < Prior colonoscopy ≤ 120 months	1 (0.3%)
Patient declined	12 (4.2%)
Lost to follow-up	86 (29.8%)
Patient frailty	2 (0.7%)
No recommendation by treating team	12 (4.2%)
Not scheduled, reason unknown	25 (8.7%)
Colonoscopy scheduled, but have not had procedure with unknown cause	103 (35.6%)
Colonoscopy performed at another hospital	15 (5.2%)
Double-contrast barium enema only performed	10 (3.5%)
Death prior to colonoscopy	1 (0.4%)

Since the frequency of polyp detection may vary according to age, we analyzed 330 patients who underwent colonoscopy into over 45 and under 45 years of age. As a result, the overall incidence of colonic polyp was 16.3% (34 patients) and 43.0% (52 patients) in patients under and over 45 years of age, respectively (*p* < 0.001). Specifically, adenoma was detected in 26 (12.4%) and 43 (35.5%) patients under and over 45 years of age, respectively (*p* < 0.001). Furthermore, hyperplastic polyp (14.9% vs. 5.7%, *p* = 0.005) and low grade dysplasia (33.9% vs. 12.4%, *p* < 0.001) were found more frequently in the age of 45 years and over. Most of all, the rate of advanced adenoma detection was statistically significantly higher in those aged 45 years or older than those under 45 years old. (5.8% vs. 1.0%, *p* = 0.009) (Table 4).

**Table 4:** Comparison of polyp detection during routine colonoscopy between under and over 45 years old

Variables	Under 45 (n = 209)	Over 45 (n = 121)	*p* value
Hyperplastic polyp	12 (5.7%)	18 (14.9%)	0.005
Adenoma			
Low grade dysplasia	26 (12.4%)	41 (33.9%)	< 0.001
High grade dysplasia	0 (0%)	2 (1.7%)	0.134
Adenocarcinoma	0 (0%)	1 (0.8%)	0.368
Advanced adenoma	2 (1.0%)	7 (5.8%)	0.009

## Discussion

The incidence rate of colon cancer detected by routine colonoscopy performed after conservative treatment of right colon acute uncomplicated diverticulitis was 0.3% (1/330 cases). The detection rate of adenoma in the whole colon was 20.9% and the frequency of advanced adenoma was 2.7%. Specifically, the adenoma detection rate (35.5% vs. 12.4%, *p* < 0.001) and the rate of advanced adenoma (5.8% vs. 1.0%, *p* = 0.009) were higher in those over 45 years old.

Clinically, the features of right colon diverticulitis are different from those of left colon diverticulitis. Specifically, compared to left colon diverticulitis, right colon diverticulitis tends to occur at a younger age, with a lower Hinchey stage, and a lower rate of recurrence (3.1% vs. 17.9% for left colon diverticulitis) [11]. Based on these facts, and considering the high diagnostic yield of CT imaging and the development of accurate pathological finding for colon cancer, the utility of routine colonoscopy in patients after treatment of acute right colon diverticulitis, in the absence of complications, such as perforation, abscess formation, and/or obstruction, has been questioned [12]. It has also been questioned if the incidence of colorectal cancer at younger ages is indeed more common on the left than on the right colon [6].

Unlike left colonic diverticulitis, which is common in Westerners, right colonic diverticulitis is more common in the Asian population, and as mentioned above, the clinical manifestations are generally milder [4]. However, while there are numerous reports on the various management strategies for left colonic diverticulitis in the West, there are few studies on right colonic diverticulitis. Above all, reports on colonoscopic evaluation of right colonic diverticulitis remain insufficient. Therefore, our results regarding colonoscopic evaluation for uncomplicated right colonic diverticulitis could serve as a useful guideline for physicians treating Asian populations.

Several studies have been published on the effects of routine colonoscopy after conservative treatment of acute uncomplicated diverticulitis. Among 205 patients who underwent colonoscopy or CT colonoscopy for all colonic diverticulitis regardless of the part, Westwood et al. reported a detection rate of 9.3% for adenomas and 0.5% for colorectal cancer [13]. Additionally, Horesh et al. reported a rate of malignant findings of 1.6% among 310 patients for all colonic diverticulitis who underwent colonoscopy. Of specific clinical relevance is the finding that there was no incidence of adenocarcinoma of the colon on follow-up colonoscopy after uncomplicated colon diverticulitis among patients younger than 50 years of age [14]. However, the majority of this evidence included only patients with left colon diverticulitis. In fact, to the best of our knowledge, only two previous studies addressed right colon diverticulitis [15, 16]. In their study of 109 patients with right-sided colon diverticulitis, Hashimoto et al. did not identify any cases of colorectal cancer, with a rate of advanced adenoma of 6.4% (7/109 cases) and non-advanced adenoma of 21.1% (23/109 cases) [16]. Chan et al. reported on 27 patients with right colon diverticulitis, with no incidence of colorectal cancer or advanced adenoma identified [15]. However, both of these studies included a small number of patients. By contrast, our study included 330 patients, a relatively large sample size. Similar to previous findings, adenoma and cancer detection rates were very low. Of significance was our finding that adenoma were identified only in the right colon.

This study was based on Korean. According to a previous population-based study about the prevalence of colorectal adenomas in asymptomatic Korean men and women published in 2014 [17], the prevalence of colorectal adenomas and advanced adenomas were 34.5% and 3.1%, respectively. Especially in subgroup analysis for under < 50 years old, although the adenoma detection rate was 20.6–24.4%, similar to that of this study (20.9%), the rate of advanced adenoma detection was fairly higher in our study (2.7%) than previous study (1.1–1.7%). This suggests that patients with right uncomplicated colonic diverticulitis are more likely to have advanced colon adenoma, so routine colonoscopy should be performed.

The adenoma detection rate (ADR) has been associated with the interval risk of colorectal cancer [18]. The ADR can be used as a colonoscopy quality indicator, with an ADR of < 20% being associated with a 10-fold increase in the interval cancer risk [19]. The ADR in our study, which included only patients with right colon diverticulitis, was 20.9%. We consider this rate to be appropriate for our study as our primary outcome was the detection rate of colon cancer.

In the previous literature, it reported that complicated diverticulitis is more likely to be associated with colon cancer than uncomplicated diverticulitis [9]. Therefore, our study also included complicated diverticulitis and tried to compare it with uncomplicated diverticulitis. However, the number of patients with complicated diverticulitis (n = 47) was smaller than previous study (n = 172). Of the 47 patients, 8 underwent emergency surgery and all patients were diagnosed with diverticulitis after surgery. In addition, only 21 out of 39 patients underwent routine colonoscopy, of which no colon cancer was found. (Additional file 1: Tables S1, S2). Therefore, complicated diverticulitis was excluded from our study.

### Limitation

The limitations of our study need to be acknowledged. Foremost, this is a retrospective study, with no knowledge of the outcomes of colonoscopy surveillance for patients who did not undergo follow-up colonoscopy. We do note that patients who did not undergo follow-up colonoscopy tended to be younger than those who did undergo colonoscopy follow-up, although there was no statistical significance. Furthermore, the presence of colon cancer was ruled out in 10 patients who underwent barium enema and 15 who underwent colonoscopy at other hospitals; however, they were excluded because of insufficient data regarding adenoma detection. If these are included, a total of 355 patients (57.7%) would have undergone surveillance for colon cancer. This rate of surveillance is comparable to previously reported rate in review articles [20, 21]. Second, as our study is not a population based, our findings do not provide an estimate of colon cancer incidence in all patients with uncomplicated right colon diverticulitis. Third, since our study was conducted on Asian patients, it may not be applicable to non-Asian populations with right colonic diverticulitis. However, the strength of our study is the relatively large sample size which, in fact, is the largest study to date evaluating routine colonoscopy results among patients with right colon diverticulitis.

## Conclusions

In patients with acute uncomplicated right colonic diverticulitis, routine colonoscopy after conservative treatment may be necessary because it is possible to detect advanced colon adenoma, even in those younger than 45 years old, who had an adenoma incidence rate of 12.4%. Especially, for patients under 45 years of age with a family history, or patients over 45 years of age who have not undergone colonoscopy within the last 3 years, screening colonoscopy is strongly recommended, which is consistent with published guidelines [5]. It will be necessary to confirm the results of our study by collecting a larger number of patients through a multicenter or population based study.

## Supplementary Information

**Additional file 1:** Clinical characteristics and adenoma detection rate between uncomplicated and complicated diverticulitis. **Supplementary Table 1:** Clinical characteristics between uncomplicated and complicated diverticulitis. **Supplementary Table 2:** Rate of detection of adenoma during routine colonoscopy between the uncomplicated and complicated diverticulitis groups.

## Data Availability

The datasets used and/or analysed during the current study available from the corresponding author on reasonable request.

## Abbreviations

ADR: Adenoma detection rate
CT: Computed tomography
IQR: Interquartile range
