# Supplementary material for: Routine colonoscopy may be needed for uncomplicated acute right colonic diverticulitis
Source: BMC Gastroenterol. 2021 Feb 27;21:91. doi: 10.1186/s12876-021-01672-1 (PMC7913260; doi:10.1186/s12876-021-01672-1)
Supplement: Supplementary file 1 — Additional file 1: Clinical characteristics and adenoma detection rate between uncomplicated and complicated diverticulitis. Supplementary Table 1: Clinical characteristics between uncomplicated and complicated diverticulitis. Supplementary Table 2: Rate of detection of adenoma during routine colonoscopy between the uncomplicated and complicated diverticulitis groups. [file 12876_2021_1672_MOESM1_ESM.docx]

**Supplementary Table 1**. Clinical characteristics between uncomplicated and complicated diverticulitis

| Variables | Uncomplicated  (n=330) | Complicated  (n=21) | p-value |
| --- | --- | --- | --- |
| Age (years) | 40 (34–50) | 47.5 (39.8–54.5) | 0.022^*^ |
| Sex  Male  Female | 195 (59.1%)  135 (40.9%) | 11 (52.4%)  10 (47.6%) | 0.545^¶^ |
| Height | 167.5 (160–173.5)^‡^ | 165.0 (160.3–169.5)^↑^ | 0.236^*^ |
| BMI (kg/m^2^) | 23.8 (21.7-26.3)^‡^ | 22.3 (21.3-23.9) ^↑^ | 0.108^*^ |
| Duration of hospital stay (days) | 4 (4-5) | 5 (4-5) | 0.588^*^ |
| Social history  Smoking  Alcohol | 142 (43.2%)  137 (42.7%) | 8 (38.1%)  6 (30.0%) | 0.649^¶^  0.265^¶^ |
| Past medical history  Hypertension  Diabetes | 50 (15.2%)  14 (4.2%) | 3 (14.3%)  1 (4.8%) | >0.999^**^  0.611^**^ |
| Location of the acute diverticulitis  Cecum  Ascending  Hepatic flexure  Transverse | 154 (46.7%)  171 (51.8%)  3 (0.9%)  2 (0.6%) | 11 (52.4%)  10 (47.6%)  0 (0%)  0 (0%) | 0.914^¶^ |
| White blood cell count (10^3^/μL) | 11.1(9.2-12.9) ^↑^ | 11.4(8.6-12.6) | 0.994^*^ |
| C-reactive protein (mg/dl) | 3.5(1.6-6.5) ^§^ | 5.1(3.3-8.1) | 0.078^*^ |

IQR, interquartile range; BMI, body mass index.

Continuous variables are reported as the median (and IQR)

Categorical variables are reported as a count (and percentage, %)

↑ Missing data: n=1, ‡ Missing data: n=11, §Missing data: n=6

*Mann-Whitney test, **Fisher’s exact test, ¶chi-squared test

**Supplementary Table 2.** Rate of detection of adenoma during routine colonoscopy between the uncomplicated and complicated diverticulitis groups

| **Variables** | **Uncomplicated**  **(n=330)** | **Complicated**  **(n=21)** | **p-value** |
| --- | --- | --- | --- |
| Hyperplastic polyp | 30 (9.1%) | 0 (0%) | 0.237 |
| Adenoma  Low grade dysplasia  High grade dysplasia | 67 (20.3%)  2 (0.6%) | 3 (14.3%)  0 (0%) | 0.778  >0.999 |
| Adenocarcinoma | 1 (0.3%) | 0 (0%) | >0.999 |
| Advanced adenoma | 9 (2.7%) | 0 (0%) | >0.999 |
| Right-sided polyp | 46 (52.9%) ^†^ | 1 (33.3%)^‡^ | 0.332 |

† number (%) of patients in whom polyps were detected in the right colon among those with detected adenoma (total number of patients, 87)

‡ number (%) of patients in whom polyps were detected in the right colon among those with detected adenoma (total number of patients, 3)
